# Hemorrhage Versus Thrombosis: A Risk Assessment for Anticoagulation Management in Pelvic Ring and Acetabular Fractures—A Registry-Based Study

**DOI:** 10.3390/jcm14103314

**Published:** 2025-05-09

**Authors:** Christof K. Audretsch, Tina Histing, Anna Schiltenwolf, Sonja Seidler, Andreas Höch, Markus A. Küper, Steven C. Herath, Maximilian M. Menger, Working Group on Pelvic Fractures of the German Trauma Society

**Affiliations:** 1 Department of Trauma and Reconstructive Surgery, BG Trauma Center Tuebingen, Eberhard Karls University Tuebingen, 72076 Tuebingen, Germany; caudretsch@bgu-tuebingen.de (C.K.A.); thisting@bgu-tuebingen.de (T.H.); aschiltenwolf@bgu-tuebingen.de (A.S.); mkueper@bgu-tuebingen.de (M.A.K.); sherath@bgu-tuebingen.de (S.C.H.); 2 Department of Orthopedics, Trauma and Plastic Surgery, University of Leipzig, 04103 Leipzig, Germany; andreas.hoech@medizin.uni-leipzig.de

**Keywords:** hemorrhage, thrombosis, pelvic ring fracture, acetabular fracture, risk evaluation, anticoagulation

## Abstract

**Background:** The increasing incidence of pelvic ring and acetabular fractures represents a major challenge in the field of trauma surgery. Hemorrhage and thrombosis are among the most severe complications associated with these injuries. The common instability of those fractures, together with an anatomic proximity to blood vessels, increases the risk of perioperative bleeding. Vascular wall irritation during surgery additionally adds to a substantial risk for thrombotic events. Therefore, evaluating the risk for hemorrhage and thrombosis in pelvic ring and acetabular fractures is vital to identify an adequate anticoagulation management. **Methods:** The incidence of hemorrhagic and thrombotic events, as well as the association of patient characteristics with the investigated outcomes of 16,359 cases, were analyzed retrospectively using data from the German Pelvic Trauma Registry. Moreover, a risk assessment survey was conducted among traumatologists experienced in pelvic ring and acetabular surgery. The results were compared to those of the registry study. **Results:** A high rate of thrombotic events was found in the middle-age decade (41–50 years). In patients with an age ≤ 40 and >50 years, hemorrhage complications predominated. The logistic regression identified pelvic ring fractures in geriatric patients, acetabular fractures, and Injury Severity Score (ISS) ≥ 16 to be associated with bleeding complications. Factors associated with thrombosis included pelvic ring fractures in non-geriatric patients, acetabular fractures in geriatric and non-geriatric patients, ISS, and male gender. The survey demonstrated that preoperatively, the risk for hemorrhage was considered more significant. Perioperatively, however, thrombosis was regarded as more important. **Conclusions:** Separate guidelines for prophylactic anticoagulation in pelvic ring and acetabular fractures that also consider individual patient characteristics, such as age, gender, and ISS, are necessary to improve perioperative management and reduce the morbidity and mortality associated with these injuries.

## 1. Introduction

The incidence of pelvic ring and acetabular fractures is increasing due to a steadily growing elderly population [1,2]. In fact, the proportion of geriatric patients is projected to increase to 25.8% of the overall population by the year 2060 [3]. Geriatric patients suffer from comorbidities such as osteoporosis, which lead to a deterioration of the skeleton as indicated by a compromised trabecular architecture and reduced biomechanical strength [4]. Therefore, in the elderly, fractures of the pelvic ring and acetabulum are, in most cases, a result of low-impact trauma. In young patients, on the other hand, these injuries are regularly caused by high-energy trauma such as traffic accidents or falls from a high altitude [5,6].

The operative management of pelvic ring and acetabular fractures represents a great challenge for the surgeon [7]. The complex bony anatomy of the pelvis, as well as its close proximity to vital anatomic structures such as the iliac vessels and the obturator neurovascular bundle [8,9], make these interventions prone to perioperative bleeding complications. Moreover, 15 to 30% of patients suffering from high-energy pelvic fractures are hemodynamically unstable, due to extensive blood loss from the fracture site and associated injuries to major blood vessels or nearby organs [10,11]. These severe hemorrhages remain the leading cause of death in patients with pelvic fractures, with a mortality of up to 32%. In such a life-threatening hemorrhage, the bleeding source is arterial in more than 70% of cases. However, overall, the vast majority of bleedings in pelvic injuries originate from venous structures, due to injuries of the soft tissue or the presacral venous plexus [12].

Another major complication in the perioperative setting of pelvic ring and acetabular fractures is the occurrence of thrombotic events. The proximity of both the injuries and the corresponding surgical approaches to the vasculature result in an increased risk for thrombosis [13], most likely caused by micro-trauma and irritation of the vascular wall. Moreover, the immobility of the patient and the inflammatory response after surgery increase the risk for thrombogenesis [14,15]. Of note, in the absence of thrombosis prophylaxis, the incidence rate of deep vein thrombosis in pelvic fractures is as high as 61% [13,16]. Therefore, adequate anticoagulation for preventing thrombosis is of major importance. On the downside, anticoagulation may increase the risk of severe hemorrhage. Hence, a thorough evaluation of the risk profile for both hemorrhage and thrombosis in pelvic ring and acetabular fractures is vital for the perioperative management of these injuries. Previous studies have examined the incidence and management of hemorrhage or thrombosis separately, without directly comparing these major risk factors [10,17,18,19]. For this purpose, we evaluated the incidence of hemorrhagic and thrombotic events in pelvic ring and acetabular fractures and analyzed possible associations between these complications and patient- and injury-related variables such as injury severity, age, and gender. Moreover, we conducted a survey on risk evaluation among traumatologists experienced in pelvic ring and acetabular surgery and compared these results with our retrospective analysis.

## 2. Material and Methods

### 2.1. Patient Collective

**Figure 1:**
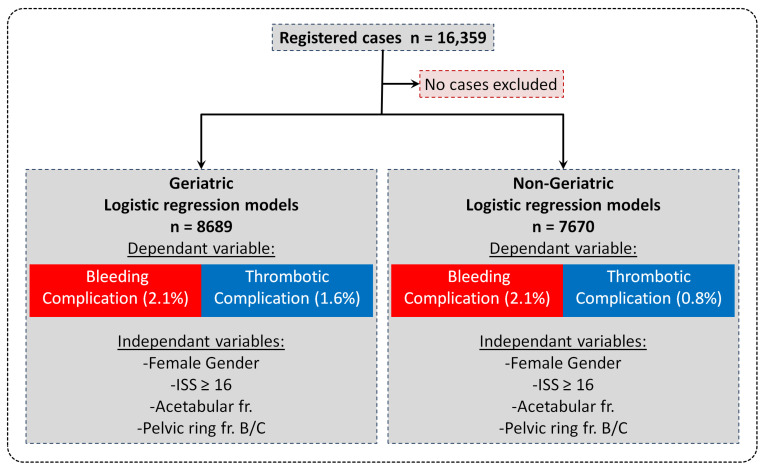
Patient selection and logistic regression models: This diagram shows the included cases (no cases excluded) and the variables of the logistic regression models.

We retrospectively analyzed data from the German Pelvic Trauma Registry (DGU Traum Registry). The registry was established in 1991 by the Pelvic Injuries Working Group of the German Association for Trauma Surgery (Deutsche Gesellschaft für Unfallchirurgie, DGU) in collaboration with the German Section of AO International to collect anonymized in-hospital data on patients with pelvic ring and/or acetabular fractures. The database is located at the Department of Trauma, Hand, and Reconstructive Surgery, University Hospital of Saarland, Homburg/Saar, Germany. Data management was performed by MEMDoc, a specialist in clinical registries at the University of Bern, Switzerland. Inclusion criteria for the registry are a pelvic ring and/or acetabular fracture and informed consent. Data on patients with pelvic fractures were collected prospectively in 30 German trauma centers between 2003 and 2017. The participating hospitals collected anonymized data on all patients treated for pelvic fractures during this period using standardized questionnaires. Informed consent was obtained from each patient included in the registry (Figure 1). Data collection was performed according to the standards approved by the Ethics Committee of the Chamber of Physicians of the Federal State of Saarland (No. 29/14). In addition, data analysis was performed in accordance with the corresponding ethics vote of the local ethics committee of the Eberhard-Karls University in Tübingen, Germany (670/2024BO2).

### 2.2. Patients’ Characteristics and Data

In this retrospective study, all 16,359 registered cases were used. The included patient records were sorted according to various factors, such as age, gender, or injury severity and checked whether either a bleeding complication or a thrombotic complication had occurred. Descriptive data were analyzed first. To assess the risk factors, the importance of the variables (age, ISS (Injury Severity Score), gender, death, acetabular fracture, pelvic ring fracture type B/C, polytrauma, and surgery) for the proportion of bleeding complications and thrombotic complications was evaluated. To identify possible confounders and adjust the OR of the factors, all 16,359 registered cases were divided into a geriatric (age ≥ 70 years) and a non-geriatric (age < 70 years) group and included in logistic regression models with the dependent binomial variables “bleeding complication” and “thrombotic complication” and the independent variables “ISS”, “gender”, “acetabular fracture”, and “pelvic ring fracture type B/C” subsequently applied. Hence, associations between these patients and injury variables and the complications can be drawn. Notably, an age of 70 years and older for geriatric patients was chosen as the cutoff age according to previous studies in the current literature [20,21,22]. A bidirectional approach was chosen for the creation of the logistic regression analysis. The initial selection of variables followed logical conclusions from the research question, including expert opinions from trauma surgeons experienced in the field of pelvic and acetabular surgery. During the iterative process, attention was paid to optimizing the Akaike information criterion (AIC). Care was also taken to avoid multicollinearity.

### 2.3. Survey

With the help of an online survey sent to the clinics participating in the pelvic registry, the treating physicians were asked about the evaluation of the risk of bleeding and thrombosis in patients with pelvic ring and acetabular fractures. Thirty-seven surgeons with extensive experience in the treatment of pelvic and acetabular fractures from the German Section for Pelvic and Acetabular Injuries of the DGU completed the online survey. This questionnaire was intended to obtain and present expert opinion, which has been used as a guideline for therapy in this regard up to now. The questions were as follows:

1. “Which risk do you estimate to be higher preoperatively (admission until surgery) for acetabular fracture (AF)?” (A) Bleeding (B) Thrombosis
2. “Which risk do you estimate to be higher perioperatively (surgery and inpatient up to 14 days after surgery) in the case of acetabular fracture (AF)?” (A) Bleeding (B) Thrombosis
3. “Which risk do you estimate to be higher preoperatively (admission until surgery) for pelvic ring fractures (PRF)?” (A) Bleeding (B) Thrombosis
4. “Which risk do you estimate to be higher perioperatively (surgery and inpatient up to 14 days after surgery) for pelvic ring fractures (PRF)?” (A) Bleeding (B) Thrombosis.

### 2.4. Statistical Analysis

For categorical variables, differences were assessed using the chi-squared test. For continuous variables, the *t*-test was used. The significance level was set at 5% (*p* = 0.05). All analyses were performed using RStudio, version 1.2.5001.

## 3. Results

### 3.1. The Impact of Patient Characteristics on Complication Frequency

**Figure 2:**
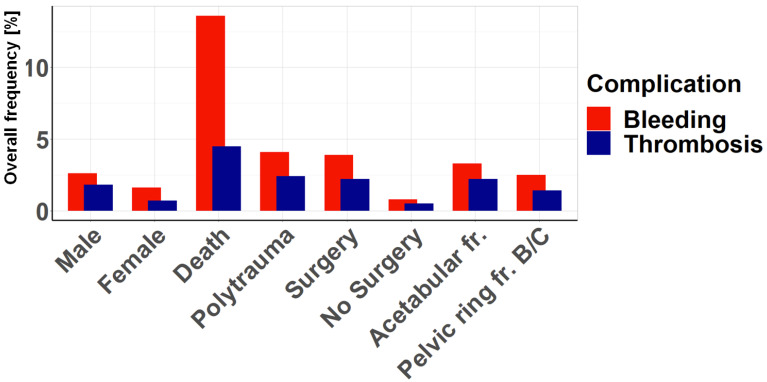
Association of the complication rate with various general factors: The overall frequency of bleeding and thrombotic complications was analyzed in relation to the variables shown. Bleeding complications were found to be the most frequent risk for each variable.

Several factors were analyzed for their association with bleeding and thrombotic complications (Figure 2). The clear association between complications and death is particularly striking. Surgery and polytrauma also show a high association with complications, whereas there are significantly fewer complications without surgical treatment. Acetabular fractures are associated with slightly more complications than pelvic ring fractures, and male patients have a higher incidence of complications than female patients. For each factor, however, bleeding complications predominate, sometimes significantly, especially in the case of death, but also in the case of polytrauma and surgical treatment.

### 3.2. Association Between Age and ISS and the Complication Rate

**Figure 3:**
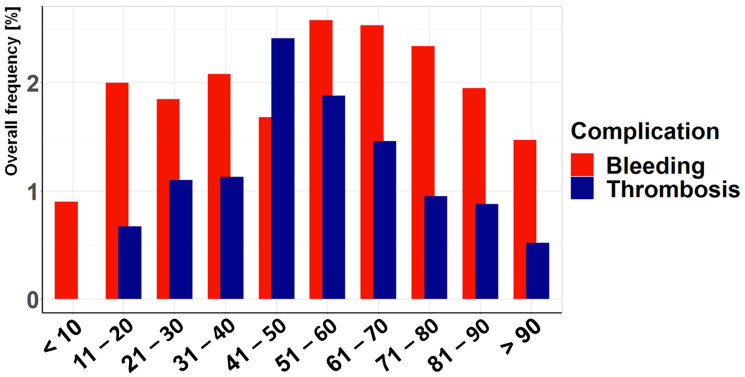
Association between age and the complication rate: This figure illustrates the overall frequency of bleeding and thrombotic complications across different age groups. While bleeding complications predominate in younger and older patients, thrombotic events peak in middle-aged patients (41–50 years). The overall complication rate declines with increasing age beyond this peak.

**Figure 4:**
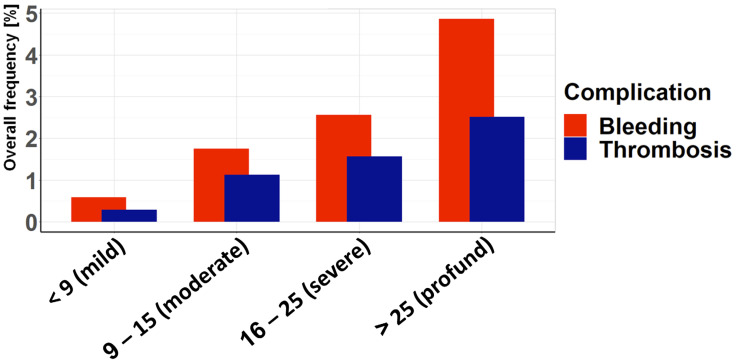
Association between ISS and the complication rate: The overall frequency of bleeding and thrombotic complications depending on the severity of injury is assessed according to the ISS. The risk of complications increases with increasing injury severity, although bleeding complications always outweigh thrombotic complications.

Patient characteristics and complications were assessed separately in non-geriatric and geriatric patients. In particular, geriatric patients are characterized by a higher proportion of females, lower injury severity, and a lower incidence of acetabular fractures and thrombotic events compared to non-geriatric patients (Table 1). The presentation of complications according to age (Figure 3) shows an increased incidence in the middle-aged range, with a decrease in older age. This pattern is even clearer when thrombotic complications are considered separately, with a particularly high risk in the middle-aged range (41–50 years). In this decade, thrombotic complications are also more common than bleeding complications. In the remaining age decades, hemorrhagic complications predominate, especially at younger and older ages. The injury severity as assessed by ISS (Figure 4) shows, as expected, an increase in both hemorrhagic and thrombotic complications with higher ISS. However, the risk of bleeding complications is consistently higher than the risk of thrombotic complications for both low and high injury severity.

### 3.3. Survey Results

**Figure 5:**
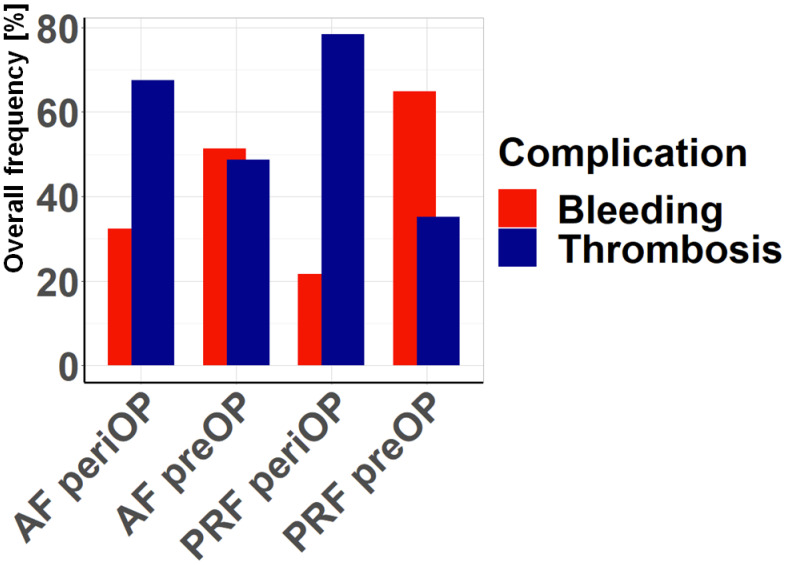
Risks perceived as more relevant: Comparison of the perceived risks of bleeding and thrombotic complications depending on PRF (pelvic ring fracture) and AF (acetabular fracture) according to the survey, both preoperatively (preOP) and perioperatively (periOP).

In the survey of members of the Pelvic and Acetabular Injuries Section of the DGU, the estimated risk of bleeding and thrombotic complications in acetabular and pelvic ring injuries was determined (Figure 5). A distinction was made between the preoperative or emergency situation and the perioperative or postoperative/inpatient situation. Preoperatively, the risk of hemorrhage is primarily seen in pelvic ring fractures. For acetabular fractures, the preoperative risk of bleeding and thrombosis is considered to be comparably high. The perceived high risk of thrombotic complications for both acetabular and pelvic ring fractures in the perioperative and postoperative/inpatient settings is striking. The thrombotic risk is considered particularly serious in the case of pelvic ring fractures.

### 3.4. Logistic Regression Analysis—Odds Ratios and Adjusted Odds

**Figure 6:**
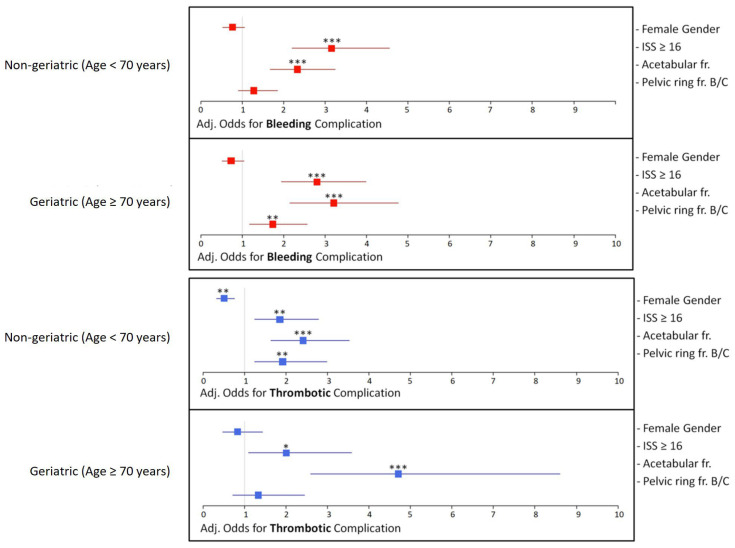
Adj. Odds for bleeding and Thrombosis: The adj. odds of different factors for thrombotic and bleeding complications are shown here, subdivided into geriatric (age ≥ 70 years) and non-geriatric (age < 70 years) patients (*p*-values are indicated as follows: *p* < 0.05 = *; *p* < 0.01 = **; *p* < 0.001 = ***).

Logistic regression was used to identify factors associated with bleeding or thrombotic complications after adjustment for other factors. For acetabular fractures, there was a significantly increased adj. odds ratio for bleeding and thrombosis, independent of age. For pelvic ring fractures B/C, the adj. odds ratio for bleeding was significantly increased in geriatric patients but not in non-geriatric patients. In contrast, the adj. odds ratio for thrombosis was significantly increased in non-geriatric patients but not in geriatric patients (Figure 6).

## 4. Discussion

In the present study, we compared the incidence of hemorrhage and thrombotic events in pelvic ring and acetabular fractures depending on patients’ characteristics such as age, gender, and ISS. Our data indicate that overall bleeding complications occur more often than thrombotic events, both in non-geriatric and geriatric patients. Interestingly, pelvic ring fractures in geriatric patients, an ISS ≥ 16, and acetabular fractures are significantly associated with bleeding complications, whereas male patients, pelvic ring fractures in non-geriatric patients, an ISS ≥ 16, and acetabular fractures were associated with thrombotic events.

Hemorrhage in pelvic ring fractures is a life-threatening event that occurs due to injuries to the vessel structure in close proximity to the ramus pubis and sacroiliac joint [23]. In acetabular fractures, bleeding complications can be caused by iatrogenic damage to the neurovascular structures during surgery [8,9,24]. Notably, our data showed that hemorrhage occurred more often compared to venous thrombosis, both in non-geriatric and geriatric patients. Only in patients aged 41 to 50 years did thrombotic events demonstrate a higher prevalence. In non-geriatric patients, the high rate of bleeding complications may be due to high-energy trauma mechanisms like traffic accidents and falls from a significant height, ultimately leading to severe damage to pivotal vessel structures in the thorax, abdomen, and pelvis [25]. Accordingly, our data showed a correlation between bleeding complications and the patient’s ISS. In geriatric patients, however, pelvic ring and acetabular fractures usually occur due to low-energy trauma [5]. The higher rate of hemorrhage in this patient collective may be explained by the abundant use of anticoagulants in the aged [5]. Notably, the diagnosis of fragility fractures of the pelvis (FFPs) is often delayed due to a postponed doctor visit and an inadequate diagnostic procedure without the use of computed tomography (CT) imaging. Thus, the identification of patients with hematoma and potentially life-threatening hemorrhage is especially challenging. Rapid and adequate diagnostics, including angiography, are vital for identifying the source of bleeding. Subsequently, the continuous monitoring of hemodynamics by blood pressure, heart rate, lactate, and hemoglobin and adequate treatment such as angioembolization or fracture stabilization are crucial for reducing morbidity and mortality [23].

In the current study, further analysis of the risk of thrombotic events demonstrated a significant association with acetabular fractures and pelvic ring fractures in non-geriatric patients. This is in line with previous studies, reporting that surgical approaches to the acetabulum bear a high risk for deep vein thrombosis, due to vascular handling procedures. Extensive retraction, fracture manipulation, malpositioned instruments, and exposure of the vascular bundle may result in compression of the vasculature, as well as micro-trauma and irritation of the vascular wall [13,26,27]. According to Virchow’s triad, these alterations can trigger blood coagulation, ultimately leading to thrombogenesis. Although life-threatening hemorrhage can be caused by iatrogenic damage to the vessel structure during surgery, bleeding complications due to damaged vessels in direct proximity to the fracture site seem more likely. In fact, pelvic ring fractures involving the ramus pubis bear the risk of damaging the obturator artery and inferior epigastric artery. Moreover, injuries involving the sacroiliac joint are prone to injury of the superior gluteal artery [23]. Information on accompanying vessel injury in acetabular fractures is sparse, and additional retrospective and prospective trials are needed. However, it may be speculated that acetabular fractures pose less risk for damaging major intrapelvic vessel structures when compared to pelvic ring fractures, while on the other hand, they are also more frequently subjected to open surgical treatment and thereby the associated risk of iatrogenic vessel injury.

Polytraumatized patients suffer from a pathophysiological inflammatory response that increases the risk for microvascular and macrovascular thrombosis by elevating circulating fibrinogen [28,29]. These findings are in line with our data, demonstrating a positive correlation between thrombotic events and patients with an ISS ≥ 16. Furthermore, our analysis showed that the female sex represents a protective factor for thrombotic events. These results are surprising since women are in general more likely to develop venous thrombosis due to the physiological hormone balance and the use of oral contraception medication [30,31]. Notably, female patients also demonstrated a lower association for bleeding complications, although the analysis did not prove to be statistically significant. Potentially, this may be associated with the higher percentage of females in the geriatric study population. However, the exact reason for the lower complication rate in female patients remains elusive. Future prospective clinical trials are needed to elucidate the gender-dependent risk during the perioperative management of acetabular and pelvic ring fractures.

Elderly people suffer from an increased risk of deep vein thrombosis due to enhanced platelet activity, increased expression of coagulation proteins such as fibrinogen as well as an elevated expression of pro-inflammatory cytokines [32]. Our analyses showed a positive correlation of pelvic ring fractures in non-geriatric patients with thrombotic events, whereas geriatric patients suffering from pelvic ring fractures showed a higher incidence of hemorrhage. In non-geriatric patients, the prolonged immobilization resulting from the injury might lead to enhanced thrombogenesis. In aged individuals, however, the use of anticoagulation drugs due to age-related comorbidities such as atrial fibrillation increases the risk for life-threatening bleeding complications [23]. Interestingly, acetabular fractures were significantly associated with both hemorrhage and thrombotic events in non-geriatric and geriatric patients. Therefore, in this type of injury, the individual patients’ characteristics, such as comorbidities and the use of anticoagulants, most likely determine which complication is more prone to develop in the perioperative setting.

Notably, the present study suffers from some limitations. Due to the retrospective study design, only associations between patients and injury characteristics could be drawn. Furthermore, additional baseline patient data, such as anticoagulant medication and comorbidities, may be crucial in determining the risk for bleeding and thrombotic events in pelvic ring and acetabular fractures. Hence, future prospective clinical trials are necessary to analyze the impact of patient characteristics on the development of hemorrhage and thrombosis during the management of these injuries.

Interestingly, the survey performed with experienced traumatologists demonstrated that most clinicians fear a thrombotic event in the perioperative management of pelvic ring and acetabular fractures. Only in the preoperative management of pelvic ring fractures was hemorrhage considered markedly more important. These expectations partially contradict our findings in the present study. Although our data showed a positive correlation between thrombotic events and acetabular fractures, these injuries are also prone to bleeding complications regardless of the patient’s age. Pelvic ring fractures in non-geriatric patients have a high risk for thrombotic events, whereas aged patients with pelvic injuries are more likely to suffer from hemorrhage. Accordingly, separate guidelines for the anticoagulant management of pelvic ring and acetabular fractures should be developed. In patients with acetabular fractures, individual characteristics such as comorbidities and the prior use of anticoagulant medication should guide the consideration of a higher dosage of anticoagulants for thromboembolism prophylaxis. In geriatric individuals with pelvic ring fractures, however, this elevated anticoagulation may increase the risk of life-threatening hemorrhage, whereas non-geriatric patients with pelvic injuries may benefit from it. Future prospective studies need to validate the results of the present study to develop reliable perioperative guidelines for anticoagulant management, also in regard to patient characteristics, such as age, gender, medication, and comorbidities.

## 5. Conclusions

Taken together, the present study demonstrates that pelvic ring fractures are significantly associated with thrombotic events in non-geriatric patients and bleeding complications in geriatric patients. Accordingly, higher prophylactic anticoagulation in pelvic ring injuries should be considered, especially in non-geriatric patients. In acetabular fractures, the patient’s age does not appear to be a significant variable influencing the incidence of hemorrhage and thrombotic events. Hence, separate guidelines for prophylactic anticoagulation in pelvic ring and acetabular fractures, including individual patient characteristics, may improve the perioperative management and reduce morbidity and mortality.

## Figures and Tables

**Table 1:** Patient, injury, and outcome characteristics of non-geriatric and geriatric patients (a = years).

**(a) Baseline Characteristics**			
	Total [%]; (*n*)	Geriatric (age ≥ 70a) [%]; (*n*)	Non-Geriatric (age < 70a) [%]; (*n*)
Age Female Gender	100% (16,359) 53.5% (8748/16,359)	46.9% (7670/16,359) 74% (5677/7670)	53.1% (8689/16,359) 35.3% (3071/8689)
**(b) Injury Characteristics**			
	Total [%]; (*n*)	Geriatric (age ≥ 70a) [%]; (*n*)	Non-Geriatric (age < 70a) [%]; (*n*)
Seriously injured (ISS ≥ 16)	32.8% (5364/16,359)	15.9% (1221/7670)	47.7% (4143/8689)
Acetabular fracture	27.8% (4547/16,359)	19.7% (1514/7670)	34.9% (3033/8689)
Pelvic ring fracture B/C	47.9% (7832/16,359)	44.4% (3407/7670)	50.9% (4425/8689)
**(c) Outcome Characteristics**			
	Total [%]; (*n*)	Geriatric (age ≥ 70a) [%]; (*n*)	Non-Geriatric (age < 70a) [%]; (*n*)
Bleeding Complication	2.1% (343/16,359)	2.1% (160/7670)	2.1% (183/8689)
Thrombotic Complication	1.2% (200/16,359)	0.8% (65/7670)	1.6% (135/8689)

## Data Availability

The data supporting the conclusions of this article will be made available by the authors on reasonable request.
